# Regional Lymph Node Metastasis in Sebaceous Carcinoma of the Head and Neck: A Systematic Review and Meta-Analysis

**DOI:** 10.3390/cancers18091424

**Published:** 2026-04-29

**Authors:** Talia A. Wenger, Margaret Nurimba, Marta Kulich, Mark S. Swanson

**Affiliations:** 1 Keck School of Medicine, University of Southern California, Los Angeles, CA 90033, USA; tawenger@usc.edu; 2 Department of Otolaryngology-Head and Neck Surgery, The Mount Sinai Hospital, New York, NY 10029, USA; margaret.nurimba@mountsinai.org; 3 Department of Otolaryngology-Head and Neck Surgery, University of Chicago, Chicago, IL 60637, USA; 4 Department of Otolaryngology-Head and Neck Surgery, University of California-Irvine, Orange, CA 92868, USA

**Keywords:** sebaceous carcinoma, regional lymph nodes, sentinel lymph node biopsy, metastases, head and neck, rare skin cancer, cutaneous adnexal tumors

## Abstract

**Background/Objectives**: Sebaceous carcinoma (SC) is a rare and aggressive malignancy most often arising in the head and neck. The reported rate of lymph node metastasis is variable and current clinical guidelines surrounding pre-treatment imaging and management of lymph nodes are not well defined. The aim of our systematic review and meta-analysis was to determine a pooled rate of clinically apparent and occult lymph node metastases for SC of the head and neck to inform clinical guidelines. **Methods**: Per PRISMA guidelines, systematic search of the Pubmed/MEDLINE and EMBASE databases identified studies published before October 2023 reporting regional lymph node status in adults with SC of the head and neck. Meta analysis using the random-effects model was applied to calculate the pooled proportion of subjects with lymph node metastasis. Clinical characteristics of subjects were further analyzed using chi square tests and univariate logistic regression. **Results**: Thirty-eight studies met inclusion criteria with a total of 2371 patients. The pooled prevalence of regional lymph node involvement, including clinically apparent and occult disease, was 16% (95% CI 13–18%, I^2^ 65%), with increased risk with increasing T stage. The pooled rate of occult lymph node metastases was 7% (95% CI 4–9%, I^2^ 68%). **Conclusions**: There is a high rate of lymph node involvement in SC of the head and neck, much of which goes undetected during initial workup and treatment. Initial workup should reflect this risk and include appropriate physical exam, imaging, consideration for sentinel lymph node biopsy, and involvement of a multi-disciplinary team.

## Simple Summary

Sebaceous carcinoma (SC) is a rare cutaneous malignancy with reported rates of locoregional metastasis of 4–33% among tumors of the head and neck. This broad range makes decisions regarding lymph node management difficult. The aim of our study was to determine a pooled rate of occult and evident lymph node metastasis among adults with SC of the head and neck to inform clinical guidelines relating to management of the lymph node basins. In a pooled cohort of 2371 patients, 16% had regional lymph node involvement, and patients with higher T stage and recurrent disease were more likely to have lymph node metastases. Seven percent of patients had occult metastases. Given this high risk of lymph node involvement, patients with SC of the head and neck should receive care from a multidisciplinary team that can provide an appropriate head and neck exam, imaging, consideration for sentinel lymph node biopsy, and treatment recommendations.

## 1. Introduction

Sebaceous carcinoma (SC) is a rare but aggressive cutaneous carcinoma arising from sebaceous adnexal structures of the skin [1]. SC usually arises in the head and neck, most commonly around the eyelids [2,3,4,5,6,7]. SC is rare, but with increasing incidence in the United States [8]. The rate of metastasis of SC of the head and neck is variable with reports from 4 to 33% and is most commonly to the regional lymph nodes [9,10,11,12,13,14,15]. Metastasis is associated with poor clinical outcomes; involvement of preauricular or cervical lymph nodes is associated with a 50 to 67% 5-year disease-specific mortality rate in periocular SC [16,17]. The mainstay of treatment of the primary site is surgery, either via wide local excision or Mohs micrographic surgery, though some patients require radiation and complex facial reconstruction for more advanced disease [18,19,20,21]. Lymph node metastases are often treated with surgery and adjuvant radiation, but a clinical question remains about the appropriate workup and management of lymph nodes without clinically apparent metastases.

As SC is rare and data regarding outcomes are limited, recommendations for initial work up are vague. Current clinical guidelines developed by the Committee on Invasive Skin Tumor Evidence-Based Recommendations (CISTERN) recommend to consider imaging in patients presenting with palpable lymphadenopathy or a tumor with high-risk features and to consider sentinel lymph node biopsy (SLNB) for periocular tumors stage T2c or higher [19]. Prior reviews have focused on treatment modalities for the primary tumor, but have not established the rate or treatment of lymph node metastasis, which is a major contributor to morbidity and mortality in SC [22]. In practice, imaging is variable and SNLB is not routinely done. While uncommon, even early-stage SC have been known to metastasize to head and neck lymph nodes [23]. Few large-volume studies exploring the rate of lymph node involvement in SC exist, thus hindering clinical decision-making ability. Similarly, there are no data regarding the rate of occult metastases, cancer cells that have spread form the primary site to lymph nodes but are too small for detection by standard routine methods. SNLB is often utilized for tumors with a high rate of occult metastasis to aid in early detection and treatment of metastatic disease, but due to limited data, is rarely utilized for SC. Understanding the rate of occult metastases would inform the potential survival benefit of a more aggressive and systematic initial workup for SC.

We conducted a systematic review and meta-analysis to determine the pooled rate of lymph node involvement in patients with SC of the head and neck based on T stage, disease time course, and other clinical factors. We aim to determine the frequency of lymph node involvement in SC patients, and in which clinical scenarios nodal metastasis is most common to inform clinical guidelines with regard to head and neck imaging, lymph nodes biopsies, and inclusion of an otolaryngologist in patient management.

## 2. Materials and Methods

### 2.1. Design and Search Strategy

**Figure 1:**
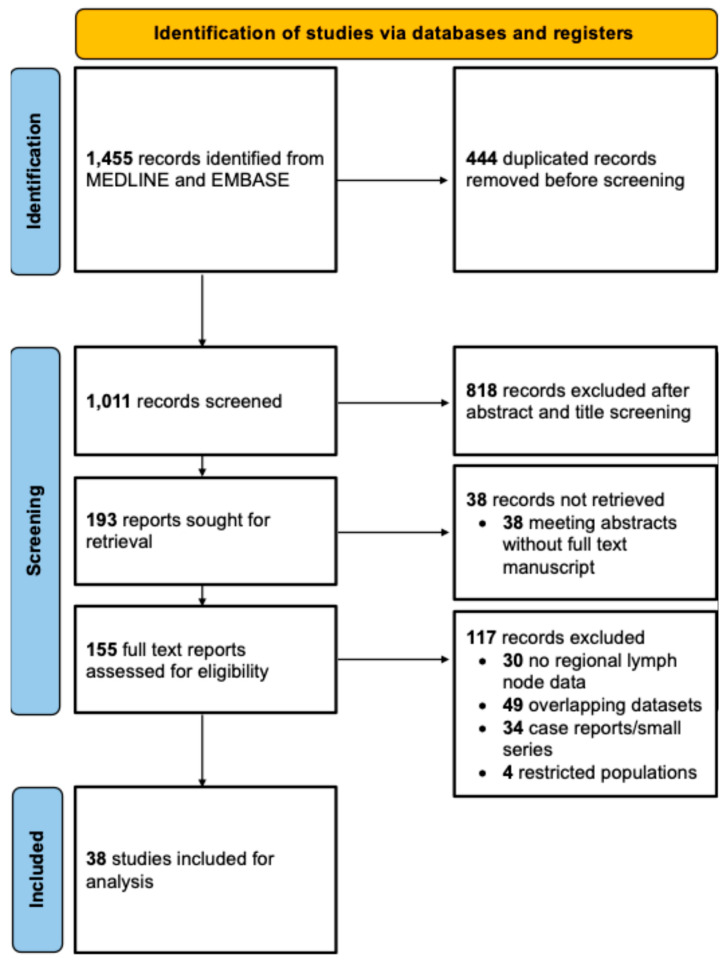
PRISMA flow diagram for study selection.

This study was conducted according to the Preferred Reporting Items for a Systematic Review and Meta-analysis (PRISMA) guidelines depicted in Figure 1. The PRISMA checklist is available in Supplementary Table S1. This study was registered with the international prospective register of systematic reviews, PROSPERO [24].

A systematic search was done using PubMed/MEDLINE and EMBASE databases to identify English language studies published before 1 October 2023 reporting regional lymph node status in adults with SC of the head and neck. Search terms used are presented in Table 1.

### 2.2. Study Selection

After removal of duplicates and screening of titles/abstracts, two authors (MK and MN) independently performed full-text reviews to assess for inclusion eligibility. Disagreements were resolved by a third independent reviewer. The following exclusion criteria was used: no regional lymph node data, same subject pool as other included studies, limited populations (such as non-representative subgroups), non-head-and-neck primary site of SC, registry or database studies (to avoid double counting of cases previously reported in case reports and series), case reports or small series < 10 subjects, and conference abstracts and letters. Study characteristics are available in Supplementary Table S2.

### 2.3. Statistical Analysis

Meta-analysis using the random-effects model was applied to calculate the pooled proportion of subjects with lymph node metastasis using thirty-eight studies. Sixteen studies that included patients with lymph node metastases at the time of initial presentation were eligible for subgroup analyses for pooled proportions. Twenty-five studies documented the presence of lymph node metastases either at presentation or at the time of disease recurrence and were analyzed via subgroup analyses for pooled proportions. Twenty-four studies documented location of recurrence (primary site versus nodal) and were used for subgroup analysis to determine the proportion of occult metastases. In this analysis, occult metastasis is defined as recurrence in regional lymph node(s) only with no recurrence at the primary site following treatment of the primary disease. Pooled proportions were calculated using a random-effects meta-analysis of binomial data applying the Freeman–Tukey double arcsine transformation with between-study variance estimated using restricted maximum likelihood (REML). Heterogeneity was assessed using Cochran’s Q test and quantified using I^2^ statistic. Publication bias and small-study effects were evaluated using funnel plot and Egger’s test. Galbraith plot was used to identify outlier studies (Supplementary Figure S1). Moderate heterogeneity was observed (I^2^ 57.3%, Q = 89.63, *p* < 0.001). Egger’s test did not demonstrate significant small-study publication bias (*p* = 0.27). Categorical variables (T stage vs. nodal metastasis) were further analyzed using a chi square test. The association between T stage and nodal metastasis was evaluated using logistic regression. T stage was analyzed both as an ordinal variable (T1–T4, modeled as a continuous predictor to estimate the odds ratio per one-stage increase) and as a categorical variable with T1 as the reference group. Odds ratios (ORs) with 95% confidence intervals (CIs) were reported. Statistical significance was defined as *p* < 0.05. All data analysis was carried out using Stata V17.0 (College Station, TX, USA).

## 3. Results

Study demographics are presented in Table 2. A total of thirty-eight studies met the criteria for inclusion in the meta-analysis, with a wide geographic distribution of study cohorts [15,25,26,27,28,29,30,31,32,33,34,35,36,37,38,39,40,41,42,43,44,45,46,47,48,49,50,51,52,53,54,55,56,57,58,59,60]. Most studies that were included were retrospective in nature. A total of 2371 patients were included with weighted mean age of 64.8 (SD 5.04) years. Of those studies specifying the primary site of the disease (2337 patients), 97.3% of sebaceous carcinoma originated in the periorbital region, while 2.7% were extraocular. A total of 13 studies included tumor and nodal staging distributions, and most patients presented with T2 (58.2%) or T3 (26.4%) stage disease. The pooled percentage of patients all types of node metastases is stratified by primary disease site and tumor staging in Table 3.

The proportion of patients with nodal metastasis differed significantly across T stages (χ^2^(3) = 214.7, *p* < 0.0001). Among patients with T1, T2, T3, and T4 tumors, 4.35%, 8.18%, 36.0%, and 58.1% had lymph node metastases, respectively. In univariable analysis, each incremental increase in T stage conferred a 4.39-fold increase in odds of nodal metastasis (95% CI 3.48–5.54; *p* < 0.001). Table 4 presents univariate logistic regression analysis. When modeled categorically with T1 as the reference, T3 and T4 disease were significantly associated with increased odds of nodal metastasis (T3 vs. T1: OR = 12.4, 95% CI 5.31–28.9; T4 vs. T1: OR = 30.5, 95% CI 11.7–79.6). T2 did not reach statistical significance (OR 1.86, 95% CI 0.831–4.62).

**Figure 2:**
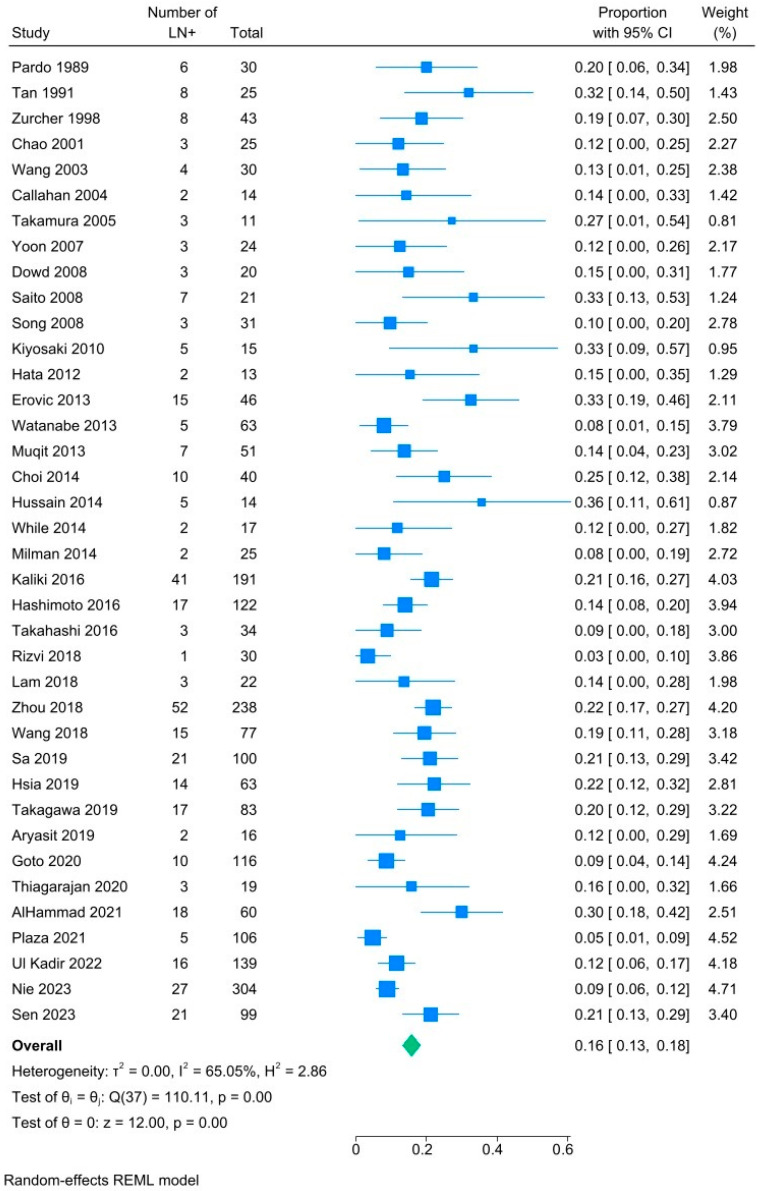
Summary forest plot analysis of overall rate of lymph node metastases for patients with sebaceous carcinoma [15,25,26,27,28,29,30,31,32,33,34,35,36,37,38,39,40,41,42,43,44,45,46,47,48,49,50,51,52,53,54,55,56,57,58,59,60,61].

A total of thirty-eight studies reported regional nodal metastases in patients with SC. The overall rate of regional lymph node involvement is assessed via forest plot in Figure 2. The pooled prevalence of nodal metastases, including those at initial presentation and upon recurrence, was 16% (95% CI, 13–18%, I^2^ 65%).

**Figure 3:**
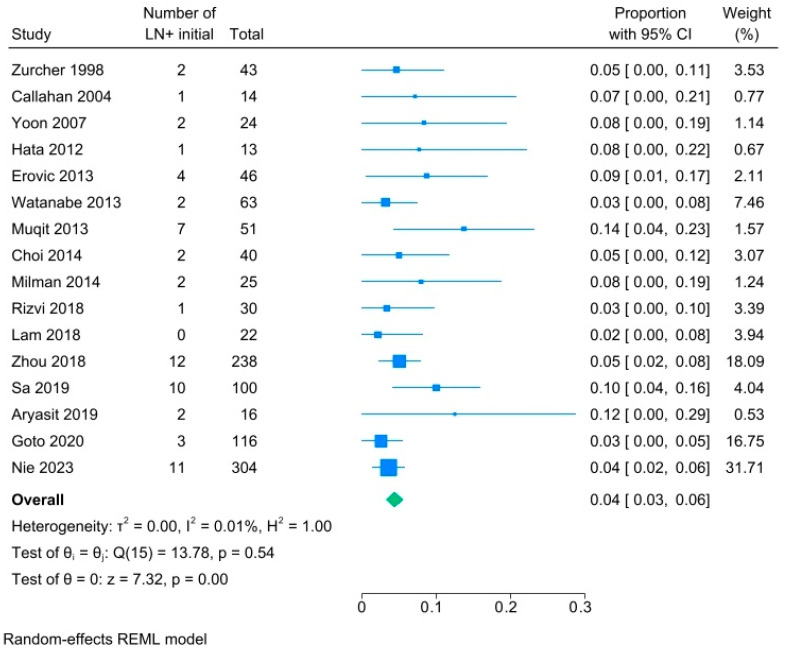
Subgroup forest plot analyses of rates of lymph node metastases at time of initial presentation for patients with sebaceous gland carcinoma [15,25,29,31,33,38,39,40,41,42,45,48,49,53,54,59].

**Figure 4:**
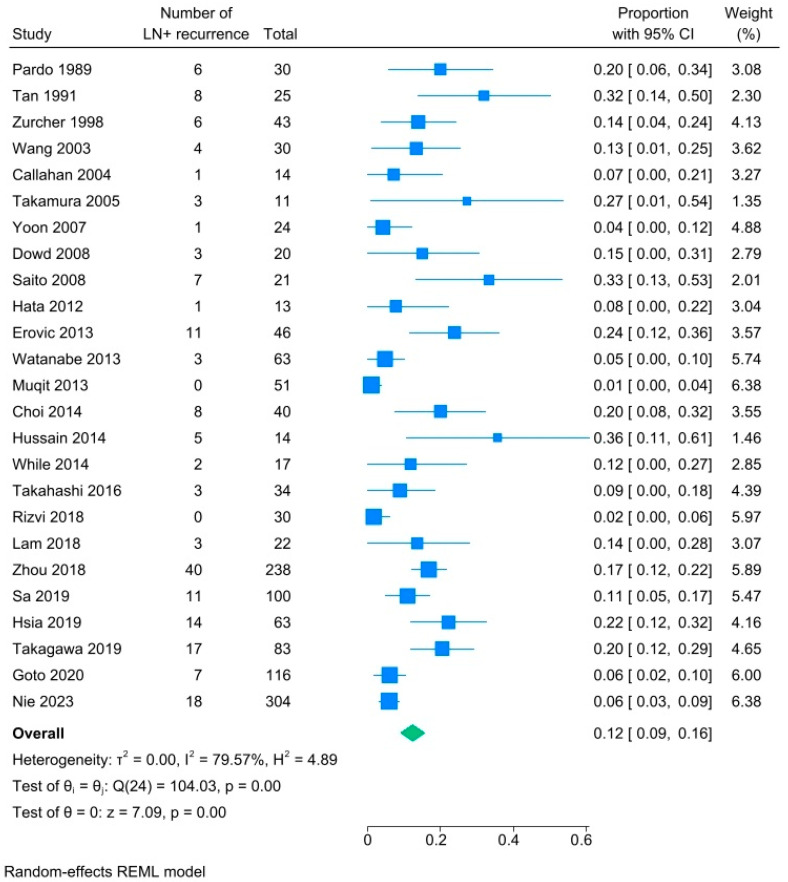
Subgroup forest plot analyses of rates of lymph node metastases at time of disease recurrence for patients with sebaceous carcinoma [15,25,27,28,29,30,31,32,33,34,35,38,39,40,41,42,43,44,47,48,49,51,52,54,59].

Subgroup analyses were performed for those studies that specified the time course of regional nodal metastases. Figure 3 demonstrates that the pooled prevalence of regional nodal metastases at the time of initial diagnosis of SC was only 4% (95% CI, 3–6%, I^2.^ 0.01%). Figure 4 demonstrates that the pooled prevalence of regional nodal metastases among patients who presented with any type of recurrence, including at the primary site and/or in the lymph nodes, following treatment of the primary tumor was 12% (95% CI 9–16%, I^2^ 79%).

**Figure 5:**
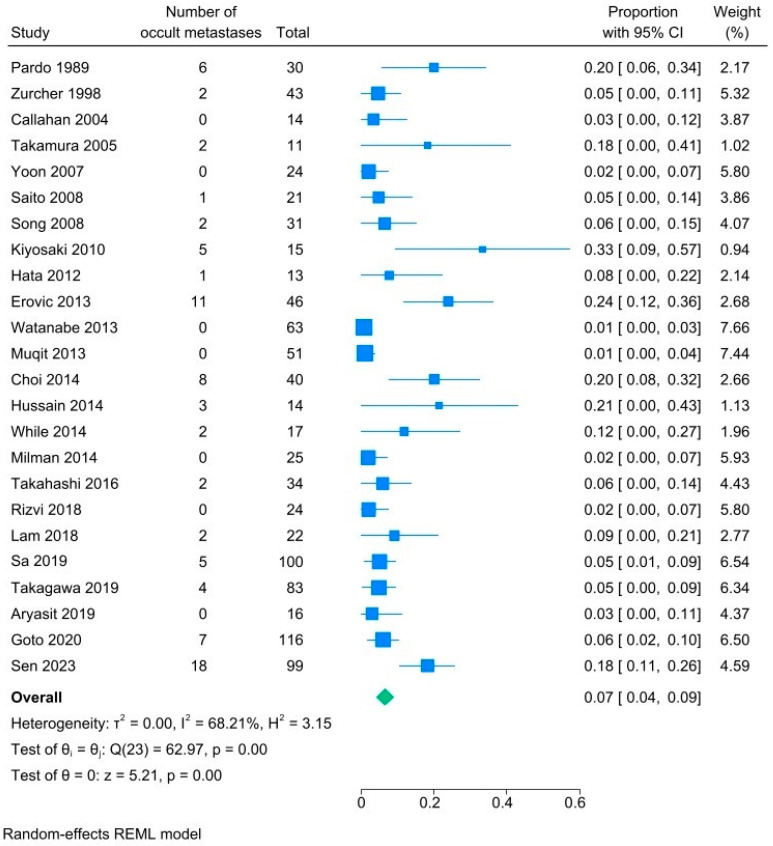
Subgroup forest plot analyses of rates of occult lymph node metastases at time of disease recurrence for patients with sebaceous carcinoma [15,27,29,31,32,33,35,36,37,38,39,40,41,42,43,44,45,47,48,49,52,53,54,60].

An additional subgroup analysis was performed for those studies that specified the location of recurrence: primary site, regional lymph node, or both. Figure 5 demonstrates that the pooled prevalence of occult metastases was 7% (95% CI 4–9%, I^2.^ 68%). This represents patients that presented with recurrence only in the regional lymph nodes, without recurrence at the primary site. These patients had occult metastases—cancer cells in the regional lymph nodes that went undetected at the time of primary work up and treatment but later became clinically evident. Among patients with occult metastases, only 11 had initial T-stage available. Of these, 7 (63.6%) were T2, 3 (27.3%) were T3, and one was T4 (9.10%).

## 4. Discussion

This systematic review and meta-analysis is, to the best of our knowledge, the largest volume study of pooled data regarding the rate of lymph node involvement in sebaceous carcinoma of the head and neck. Of the 38 articles reviewed with a total of 2371 patients, lymph node involvement was found to occur in 16% of all cases, with increasing risk for advanced T stage. The reported rates of recurrence in sebaceous carcinoma are highly variable ranging from 4 to 33% with only 5.7% of patients presenting at initial presentation with nodal metastasis [11,16,21,26,40,62,63]. High rates of recurrence indicate an opportunity to improve initial work up to ensure detection of the full scope of disease.

While this study found that most cases with lymph node metastasis were in patients with high stage tumors (T3 and T4), and that increasing T stage is associated with increased risk of lymph node metastasis, T1 and T2 tumors also had small but clinically meaningful levels of nodal involvement (4% and 8%, respectively). This is almost certainly an underestimate due to diagnostic bias, as imaging of the neck is not currently recommended for most patients. Multiple studies have found that T1 and T2 tumors carry a small but significant risk of nodal metastasis [47,64,65]. Morawala et al. [64] found a 5% and 9%, respectively, 5-year risk of lymph node metastasis in T1 and T2 sebaceous carcinomas. A 2016 case series by Takahashi et al. [47] revealed that a patient with a T2 tumor without high risk factors and a complete resection with clear margins proceeded to have metastasis to the parotid. Even low-grade tumors carry the risk for nodal metastasis, further highlighting the importance of lymph node screening for all patients with sebaceous carcinoma. Notably, there is heterogeneity in the AJCC edition used for TNM staging data in this meta-analysis, as studies utilized different guidelines, which may limit the generalizability of this finding. Such heterogeneity, and that many studies did not report T-stage, limited this analysis.

Most notably in this study, 7% of patients had occult metastases. This suggests a significant risk of occult nodal disease, as these patients did not have local recurrence but still presented with nodal disease following initial treatment. These patients likely had lymph node metastases that went undetected, and thus untreated, in the initial work up. Had the nodal involvement been detected earlier, these patients may have undergone more aggressive initial treatment with potential for improved outcomes. As many patients do not receive neck imaging or SLNB as part of their initial work up for sebaceous carcinoma, it is impossible to know if these occult metastases were too small to detect by clinical exam and imaging, or if these screening tools were never utilized at all. The definition of occult disease used in this study is recurrence-based, rather than relying upon SLNB data which can show microscopic nodal disease that is not apparent clinically or on imaging. As such, this definition is narrower and is likely an underestimate of the true rate of occult disease.

Current guidelines for management of sebaceous carcinoma were developed via a consensus-based multidisciplinary conference of experts [19]. These guidelines recommend considering imaging for periocular tumors stage T2c and higher or tumors with high-risk features, such as poorly differentiated pathology, pagetoid spread, or perineural invasion. Lymph node sampling via fine-needle aspiration or core needle biopsy is recommended only in the case of enlarged lymph nodes identified on exam. Such recommendations are based on a paucity of data regarding rates of regional lymph node metastasis and occult metastases. There is little guidance regarding SNLB to detect occult metastatic disease.

Nevertheless, metastasis to the lymph nodes is a poor prognostic factor in sebaceous carcinoma [1,15,66]. Zhou et al. found that for patients with lymph node involvement upon initial presentation, survival rate was just 50% with a median survival time of 28.0 months [25]. Given the high rates of recurrence, poor outcomes associated with recurrence, increasing risk of lymph node metastasis with T stage, and the finding that 7% of patients have occult metastases, all patients with sebaceous carcinoma of the head and neck should receive a thorough head and neck lymph node exam and be offered neck imaging. Patients with T3 and T4 tumors, and some patients with T2 tumors, may benefit from SNLB upon initial treatment. Risks and benefits should be discussed with patients individually, with the understanding that there is a significant risk of occult metastasis in sebaceous carcinoma and that lymph node involvement is associated with poorer prognosis.

Though early case studies support the use of SNLB in periocular sebaceous carcinoma, the effect of positive sentinel lymph node biopsy on survival is unknown [67,68,69]. Thus, SNLB is not routinely recommended in management of sebaceous carcinoma, though it can be considered for tumors stage T2c and higher. Among sebaceous carcinoma, SNLB has a positivity rate of 7.4–16% and a false negative rate of 7.14–26.7% [15,70,71,72]. In melanoma of the head and neck, in which SNLB is recommended for all tumors stage T1b and higher, the rate of lymph node positivity is 12.9–20% and the rate of false negatives is 9.4–34.5% [73,74]. Despite high rates of false negative, SNLB provides a significant survival benefit to patients with melanoma [75]. Given the efficacy of sentinel lymph node biopsy in melanoma patients, and the similarity in rate of positivity and false negatives, it is possible that SNLB could have a similarly significant survival benefit in patients with sebaceous carcinoma. In this current study, patients with T3 and T4 disease have a 36.0%, and 58.1% risk of presenting with lymph node metastases. Due to the high risk of nodal recurrence, these patients are reasonable candidates for pre-operative PET/CT and SNLB to accurately stage their disease and escalate treatment as necessary. Given the lack of data showing survival benefit of SLNB in sebaceous carcinoma, these decisions should be individualized given a patient’s risk, health status, and preferences. Additionally, 8.18% of patients with T2 disease present with lymph node metastasis, and though the rate of occult metastasis per T stage cannot be determined, at least some of that risk is attributed to T2 disease. These patients, especially those with high-risk features, may be considered for SNLB as well. In these patients especially, the potential benefits of SNLB should be weighed against the limitations, such as the rate of false negatives and the need for appropriate imaging and/or multidisciplinary team to effectively identify the sentinel lymph node. Additionally, the risk of complications, such as lymphedema, seroma or hematoma, infection, nerve injury, or anaphylaxis to blue dyes should be considered [76].

This study is limited by its retrospective nature and study-level heterogeneity. Study heterogeneity in this meta-analysis was moderate and significant due to differences in design, staging, and reported data among included studies. This may limit the generalizability of our findings. Subgroup analyses were required as staging and recurrence data were limited in some studies. Additionally, staging data is mixed as they were pulled directly from papers which utilized different AJCC and TNM staging criteria. Future research should focus on developing a prospective study of lymph node involvement in patients with sebaceous carcinoma of the head and neck, which would allow for less diagnostic bias in the results. A prospective study delineating the efficacy of early imaging and SLNB in improving survival or preventing recurrence would be especially valuable in clarifying imaging and treatment guidelines. Such studies may be particularly valuable in patients with stage T2 sebaceous carcinoma, where the risk of occult disease is more nebulous. Given the rarity of SC, multicenter cohorts or prospective registries are reasonable approaches to ensure adequate study size. Such study designs may also ensure a diverse and more representative patient cohort studied under similar circumstances, as one of the major limitations in this study is the heterogeneity of study populations and methods.

## 5. Conclusions

Our meta-analysis found a significant rate of lymph node involvement in sebaceous carcinoma, with significantly increased risk for T3 and T4 tumors. While the risk of nodal metastasis is greatest in high stage tumors, there is a notable risk among all tumor stages. Given the poor prognosis associated with nodal metastasis and the need for aggressive treatment, diagnosis of sebaceous carcinoma of any T stage warrants evaluation of the lymph nodes through physical exam and head and neck imaging. Given the high rate of occult metastasis and increasing risk of lymph node metastasis with increasing T stage observed in this study, SLNB should be strongly considered in patients with T3 and T4 tumors and may be appropriate in those with T2 tumors with high-risk features. Patients would benefit from a multi-disciplinary team to ensure full evaluation of lymph node involvement via physical exam, imaging, and SLNB and to promote best possible outcomes.

## Figures and Tables

**Table 1:** Study search criteria utilized in the meta-analysis.

Database	Search Criteria
PubMed	(“sebaceous gland neoplasms”[MeSH Terms]) OR “sebaceous carcinoma” OR “sebaceous gland carcinoma” OR “sebaceous adenocarcinoma” OR “meibomian gland carcinoma”) AND ((lymph node*) OR (lymph*) OR (metastas*)) AND (“english”[Language])
Embase	(“sebaceous carcinoma” OR “sebaceous gland carcinoma” OR “sebaceous adenocarcinoma” OR “meibomian gland carcinoma”) AND ((Lymph nod*) OR (lymph*) OR (metastas*)) AND [english]/lim

**Table 2:** Summary of patient demographics from studies included in meta-analysis.

Variable	*n*	%
Study Region		
North America	9	23.7
Southeast Asia	2	5.3
Europe	3	7.9
East Asia	17	44.7
Australia	1	2.6
South Asia	4	10.5
West Asia	2	5.3
Total	38	
Study Design		
Retrospective	37	
Prospective	1	
Total	38	
Age (weighted mean, SD)	64.8 (5.04)	
Sex		
Male	992	41.8
Female	1353	57.1
Not Specified	26	1.10
Total	2371	
Primary Site		
Periorbital *	2274	95.9
Extraocular	63	2.65
Not specified	34	1.43
Total	2371	
Tumor Stage		
T1	138	5.82
T2	758	32.0
T3	344	14.5
T4	62	2.61
Not specified	1069	45.1
Total	2371	

* Periorbital includes eyelid, caruncle, brow, and periorbital adnexa.

**Table 3:** Proportions of patients with sebaceous carcinoma and lymph node metastasis compared to all patients, stratified by primary site and by tumor stage.

	Total Patients		With Lymph Node Metastases	
	*n*	%	*n*	% of Total
Primary Site				
Periorbital *	2274	97.3%	381	16.8%
Extraorbital	63	2.7%	5	7.9%
Total	2337		386	16.5%
Tumor Stage				
T1	138	10.6%	6	2.6%
T2	758	58.2%	62	27.2%
T3	344	26.4%	124	54.4%
T4	62	4.8%	36	15.8%
Total	1302		228	

* Periorbital includes eyelid, caruncle, brow, and periorbital adnexa.

**Table 4:** Univariate logistic regression analysis for association between tumor stage and lymph node metastasis.

	Total Patients (*n*)	% of *n* with Metastasis	OR (95% CI)	*p*-Value
Tumor Stage				
T1	138	4.35%	(ref)	
T2	758	8.18%	1.86 (0.831–4.62)	0.125
T3	344	36.0%	12.4 (5.31–28.9)	<0.0001
T4	62	58.1%	30.5 (11.7–79.6)	<0.0001

## Data Availability

No new data were created or analyzed in this study. Data sharing is not applicable to this article.

## Supplementary Materials

The following supporting information can be downloaded at: https://www.mdpi.com/article/10.3390/cancers18091424/s1, Table S1: PRISMA Checklist, Table S2: Study Characteristics, Figure S1: Funnel and Galbraith plot for sensitivity analysis.

## Abbreviations

The following abbreviations are used in this manuscript:

SC	Sebaceous carcinoma
LN	Lymph node
PRISMA	Preferred Reporting Items for Systemic reviews and Meta-Analyses
SLNB	Sentinel lymph node biopsy
AJCC	American Joint Committee on Cancer
CISTERN	Committee on Invasive Skin Tumor Evidence-Based Recommendations
SD	Standard deviation
