# Supplementary material for: Regional Lymph Node Metastasis in Sebaceous Carcinoma of the Head and Neck: A Systematic Review and Meta-Analysis
Source: Cancers (Basel). 2026 Apr 29;18(9):1424. doi: 10.3390/cancers18091424 (PMC13162734; doi:10.3390/cancers18091424)
Supplement: Supplementary file 1 [file cancers-18-01424-s001.zip › cancers-4211490-supplementary.pdf]

Supplemental Table S1: PRISMA Checklist			
**Section and Topic**	**Item #**	**Checklist item**	**Location where item is reported**
**TITLE**			
Title	1	Identify the report as a systematic review.	Page 1
**ABSTRACT**			
Abstract	2	See the PRISMA 2020 for Abstracts checklist.	See below (supp table S1)
**INTRODUCTION**			
Rationale	3	Describe the rationale for the review in the context of existing knowledge.	Page 2
Objectives	4	Provide an explicit statement of the objective(s) or question(s) the review addresses.	Page 2
**METHODS**			
Eligibility criteria	5	Specify the inclusion and exclusion criteria for the review and how studies were grouped for the syntheses.	Page 3-4, figure 1
Information sources	6	Specify all databases, registers, websites, organisations, reference lists and other sources searched or consulted to identify studies. Specify the date when each source was last searched or consulted.	Page 3-4, table 1
Search strategy	7	Present the full search strategies for all databases, registers and websites, including any filters and limits used.	Page 3-4
Selection process	8	Specify the methods used to decide whether a study met the inclusion criteria of the review, including how many reviewers screened each record and each report retrieved, whether they worked independently, and if applicable, details of automation tools used in the process.	Page 3-4
Data collection process	9	Specify the methods used to collect data from reports, including how many reviewers collected data from each report, whether they worked independently, any processes for obtaining or confirming data from study investigators, and if applicable, details of automation tools used in the process.	Page 3-4
Data items	10a	List and define all outcomes for which data were sought. Specify whether all results that were compatible with each outcome domain in each study were sought (e.g. for all measures, time points, analyses), and if not, the methods used to decide which results to collect.	Page 4-5
	10b	List and define all other variables for which data were sought (e.g. participant and intervention characteristics, funding sources). Describe any assumptions made about any missing or unclear information.	Page 4-5
Study risk of bias assessment	11	Specify the methods used to assess risk of bias in the included studies, including details of the tool(s) used, how many reviewers assessed each study and whether they worked independently, and if applicable, details of automation tools used in the process.	Page 4
Effect measures	12	Specify for each outcome the effect measure(s) (e.g. risk ratio, mean difference) used in the synthesis or presentation of results.	Page 4-5
Synthesis methods	13a	Describe the processes used to decide which studies were eligible for each synthesis (e.g. tabulating the study intervention characteristics and comparing against the planned groups for each synthesis (item #5)).	Page 4, figure 1
	13b	Describe any methods required to prepare the data for presentation or synthesis, such as handling of missing summary statistics, or data conversions.	Page 4-5
	13c	Describe any methods used to tabulate or visually display results of individual studies and syntheses.	Page 4-5
	13d	Describe any methods used to synthesize results and provide a rationale for the choice(s). If meta-analysis was performed, describe	Page 4-5

Supplemental Table S1: PRISMA Checklist			
**Section and Topic**	**Item #**	**Checklist item**	**Location where item is reported**
		the model(s), method(s) to identify the presence and extent of statistical heterogeneity, and software package(s) used.	
	13e	Describe any methods used to explore possible causes of heterogeneity among study results (e.g. subgroup analysis, meta-regression).	Page 4-5
	13f	Describe any sensitivity analyses conducted to assess robustness of the synthesized results.	Page 4-5
Reporting bias assessment	14	Describe any methods used to assess risk of bias due to missing results in a synthesis (arising from reporting biases).	Page 4-5
Certainty assessment	15	Describe any methods used to assess certainty (or confidence) in the body of evidence for an outcome.	Page 4-5
**RESULTS**			
Study selection	16a	Describe the results of the search and selection process, from the number of records identified in the search to the number of studies included in the review, ideally using a flow diagram.	Figure 1
	16b	Cite studies that might appear to meet the inclusion criteria, but which were excluded, and explain why they were excluded.	Figure 1
Study characteristics	17	Cite each included study and present its characteristics.	Supplemental table S2
Risk of bias in studies	18	Present assessments of risk of bias for each included study.	Figure 2, 3, 4, 5
Results of individual studies	19	For all outcomes, present, for each study: (a) summary statistics for each group (where appropriate) and (b) an effect estimate and its precision (e.g. confidence/credible interval), ideally using structured tables or plots.	Figure 2, 3, 4, 5
Results of syntheses	20a	For each synthesis, briefly summarise the characteristics and risk of bias among contributing studies.	Pages 5-7
	20b	Present results of all statistical syntheses conducted. If meta-analysis was done, present for each the summary estimate and its precision (e.g. confidence/credible interval) and measures of statistical heterogeneity. If comparing groups, describe the direction of the effect.	Figure 2, 3, 4, 5
	20c	Present results of all investigations of possible causes of heterogeneity among study results.	Figure 2, 3, 4, 5
	20d	Present results of all sensitivity analyses conducted to assess the robustness of the synthesized results.	Supplemental figure S1
Reporting biases	21	Present assessments of risk of bias due to missing results (arising from reporting biases) for each synthesis assessed.	Figure 2, 3, 4, 5
Certainty of evidence	22	Present assessments of certainty (or confidence) in the body of evidence for each outcome assessed.	Figure 2, 3, 4, 5
**DISCUSSION**			
Discussion	23a	Provide a general interpretation of the results in the context of other evidence.	Pages 7-10
	23b	Discuss any limitations of the evidence included in the review.	Page 10
	23c	Discuss any limitations of the review processes used.	Page 10
	23d	Discuss implications of the results for practice, policy, and future research.	Pages 7-10
**OTHER INFORMATION**			
Registration and	24a	Provide registration information for the review, including register name and registration number, or state that the review was not	Page 3

Supplemental Table S1: PRISMA Checklist			
**Section and Topic**	**Item #**	**Checklist item**	**Location where item is reported**
protocol		registered.	
	24b	Indicate where the review protocol can be accessed, or state that a protocol was not prepared.	Pages 3-4
	24c	Describe and explain any amendments to information provided at registration or in the protocol.	None
Support	25	Describe sources of financial or non-financial support for the review, and the role of the funders or sponsors in the review.	None
Competing interests	26	Declare any competing interests of review authors.	None
Availability of data, code and other materials	27	Report which of the following are publicly available and where they can be found: template data collection forms; data extracted from included studies; data used for all analyses; analytic code; any other materials used in the review.	Available upon request

PRISMA Abstract Checklist			
**Section and Topic**	**Item #**	**Checklist item**	**Reported (Yes/No)**
**TITLE**			
Title	1	Identify the report as a systematic review.	Yes
**BACKGROUND**			
Objectives	2	Provide an explicit statement of the main objective(s) or question(s) the review addresses.	Yes
**METHODS**			
Eligibility criteria	3	Specify the inclusion and exclusion criteria for the review.	Yes
Information sources	4	Specify the information sources (e.g. databases, registers) used to identify studies and the date when each was last searched.	Yes
Risk of bias	5	Specify the methods used to assess risk of bias in the included studies.	Yes
Synthesis of results	6	Specify the methods used to present and synthesise results.	Yes
**RESULTS**			
Included studies	7	Give the total number of included studies and participants and summarise relevant characteristics of studies.	Yes
Synthesis of results	8	Present results for main outcomes, preferably indicating the number of included studies and participants for each. If meta-analysis was done, report the summary estimate and confidence/credible interval. If comparing groups, indicate the direction of the effect (i.e. which group is favoured).	Yes
**DISCUSSION**			
Limitations of evidence	9	Provide a brief summary of the limitations of the evidence included in the review (e.g. study risk of bias, inconsistency and imprecision).	Yes
Interpretation	10	Provide a general interpretation of the results and important implications.	Yes
**OTHER**			
Funding	11	Specify the primary source of funding for the review.	Yes
Registration	12	Provide the register name and registration number.	Yes

**Supplemental Table S2: Study Characteristics**				
**Study Name**	**Year of Publication**	**Type**	**Institution**	**Region**
Pardo 1989	1989	retrospective	Massechusettes General	North America
Tan 1991	1991	retrospective	Singapore General Hospital	Southeast Asia
Zurcher 1998	1998	retrospective	Moorfields Eye Hospital (London)	Europe
Chao 2001	2001	retrospective	Wills Eye Hospital (Philadelphia)	North America
Wang 2003	2003	retrospective	National Taiwan University and Far Eastern Memorial Hospitals	East Asia
Callahan 2004	2004	retrospective	Mayo Clinic (Rochester)	North America
Takamura 2005	2005	retrospective	Yamagata University Hospital (Japan)	East Asia
Yoon 2007	2007	retrospective	Yonsei University College of Medicine (South Korea)	East Asia
Dowd 2008	2008	retrospective	Nepean Hospital (Sydney, AU)	Australia
Saito 2008	2008	retrospective	Hokkaido University Hospital (Japan)	East Asia
Song 2008	2008	retrospective	University of Iowa	North America
Kiyosaki 2010	2010	retrospective	Oita University Hospital (Japan)	East Asia
Hata 2012	2012	retrospective	Yokahama City University	East Asia
Erovic 2013	2013	retrospective	Princess Margaret (Toronto)	North America
Watanabe 2013	2013	retrospective	Kyoto Prefectural University of Medicine Hospital and Seirei Hamamatsu Hospital	East Asia
Muqit 2013	2013	prospective	Oxford (UK)	Europe
Choi 2014	2014	retrospective	Seoul National Universtiy Hospital	East Asia
Hussain 2014	2014	retrospective	University of Miami (Bascom Eye institute)	North America
While 2014	2014	retrospective	Sheffield Teaching Hospitals (UK)	Europe
Milman 2014	2014	retrospective	New York Eye and Ear Infirmary	North America
Kaliki 2016	2016	retrospective	LV Prasad eye institute	South Asia
Hashimoto 2016	2016	retrospective	Kyushu University Hospital (Fukuoka, Japan)	East Asia
Takahashi 2016	2016	retrospective	Aichi Medical University Hospital (Japan)	East Asia
Rizvi 2018	2018	retrospective	Oman	West Asia
Lam 2018	2018	retrospective	Hong Kong Eye institute	East Asia
Zhou 2018	2018	retrospective	Ninth People's Hopital/Shanghai Jiao tong University (China)	East Asia
Wang 2018	2018	retrospective	Bengbu Medical College (China)	East Asia

So 2019	2019	retrospective	MD Anderson	North America
Hsia 2019	2019	retrospective	National Taiwan University Hospital	East Asia
Takagawa 2019	2019	retrospective	National Center Cancer Hospital (Tokyo)	East Asia
Aryasit 2019	2019	retrospective	Songklanagarind Hospital (thailand)	Southeast Asia
Goto 2020	2020	retrospective	Tokyo medical university hospital	East Asia
Thiagarajan 2020	2020	retrospective	Tata Memorial Centre (Mumbai)	South Asia
AlHammad 2021	2021	retrospective	Saudi Arabia, and the Orbital Unit of the University of Naples	West Asia
Plaza 2021	2021	retrospective	Ohio State	North America
Ul Kadir 2022	2022	retrospective	Bangladesh Eye Hospital and Institute	South Asia
Nie 2023	2023	retrospective	Beijing Tongren Hospital	East Asia
Sen 2023	2023	retrospective	All India Institute of Medical Sciences (New Dehli)	South Asia

Supplemental Figure S1. Funnel and Galbraith plot for sensitivity analysis

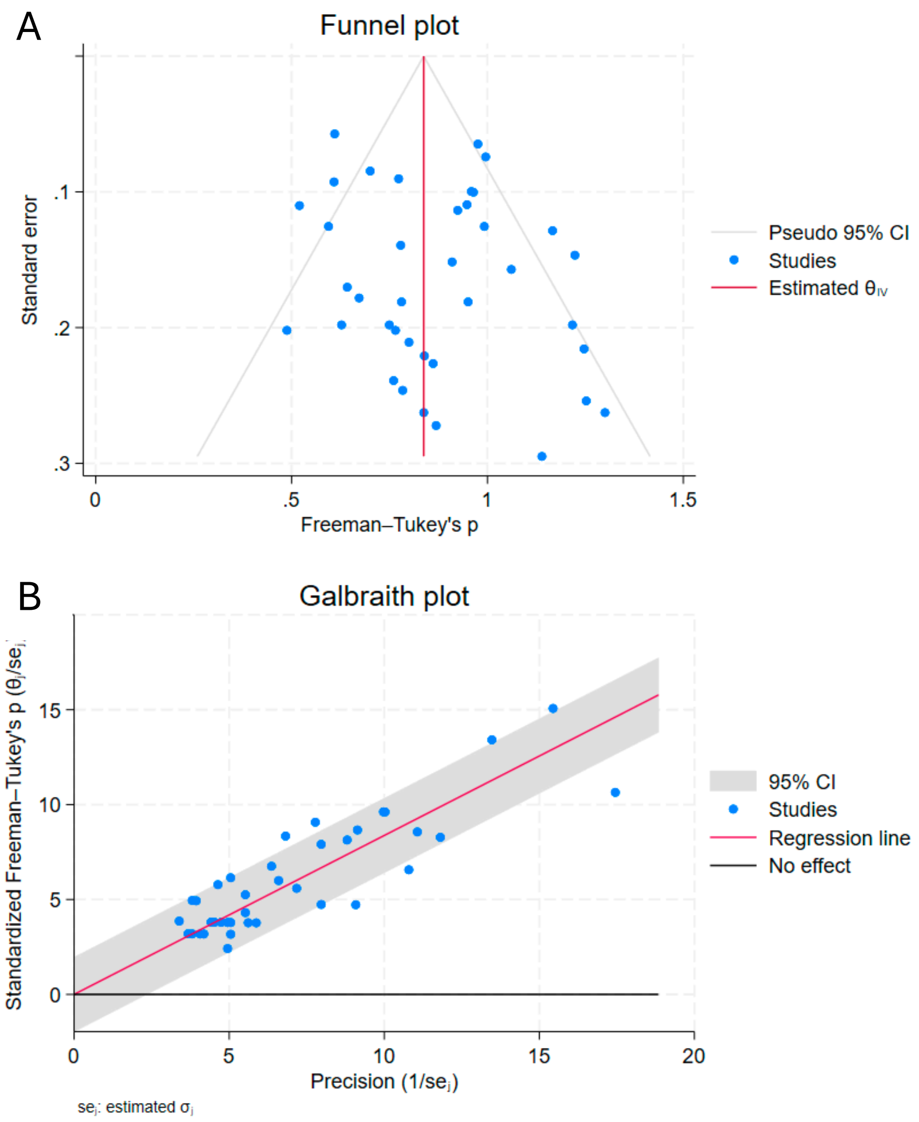

Egger's Test: $\beta = 0.64$, SE = 0.58, $z = 1.11$, $p=0.266$
